# Study on the Anti-Melanogenesis and Anti-Photoaging Effects of *Sargassum fusiforme* Polyphenols Based on Zebrafish and Cell Models

**DOI:** 10.3390/biology15161388

**Published:** 2026-08-13

**Authors:** Jiamin Lu, Mo Chen, Chuner Cai, Peimin He, Denghui Shu, Ya Zhao, Rui Jia

**Affiliations:** 1 College of Oceanography and Ecological Science, Shanghai Ocean University, Shanghai 201306, China; void159@163.com (J.L.); cecai@shou.edu.cn (C.C.); pmhe@shou.edu.cn (P.H.); 2 Shanghai Jahwa United Co., Ltd., Shanghai 200082, China; chenmo@jahwa.com.cn; 3 School of Environmental and Chemical Engineering, Shanghai University, Shanghai 200444, China; 1501034469@shu.edu.cn; 4 Marine Biomedical Science and Technology Innovation Platform of Lingang Special Area, Shanghai 201306, China

**Keywords:** *Sargassum fusiforme*, polyphenols, skin-whitening, anti-photoaging, zebrafish

## Abstract

Skin photoaging is primarily induced by ultraviolet (UV) radiation and is accompanied by extracellular matrix (ECM) degradation, abnormal pigmentation, and loss of skin elasticity. Excessive melanin synthesis represents a key cause of pigmentary skin disorders. Natural polyphenols with dual skin-whitening and anti-photoaging activities have attracted increasing attention as cosmetic ingredients. This study examined the effects of polyphenol-enriched fraction from the brown alga *Sargassum fusiforme* (SFP). Zebrafish embryos and human melanoma A375 cells were used to evaluate the depigmenting efficacy of SFP, while UVA-induced human dermal fibroblasts (HDFs) were employed to assess its anti-photoaging activity. SFP significantly decreased melanin levels in both models in a dose-dependent and reversible manner by suppressing tyrosinase activity. In terms of anti-photoaging effects, SFP markedly suppressed UVA-induced matrix metalloproteinases (MMPs) expression and elevated key ECM components, including type I collagen, elastin, and hyaluronic acid. In conclusion, SFP exerts dual beneficial effects by attenuating melanogenesis via tyrosinase inhibition and alleviating photoaging damage by suppressing MMPs to protect ECM. These results provide a scientific foundation for the use of SFP as a dual-purpose natural cosmetic ingredient with significant potential in the cosmetics sector.

## Simple Summary

Prolonged sun exposure causes ultraviolet damage to the skin and accelerates aging. It also stimulates melanin overproduction, leading to dark spots and uneven skin tone. Natural polyphenols are being studied for their potential in skin protection. This study evaluated a polyphenol-enriched fraction from the brown alga *Sargassum fusiforme* for its effects on melanin reduction and the prevention of skin aging. The results showed that this extract effectively inhibited the key enzyme involved in melanin synthesis, thereby reducing pigment accumulation. Additionally, it reduced the degradation of collagen and elastin, which are vital for preserving skin firmness and suppleness, and lessened UV-induced damage to skin cells. This study suggests that *S. fusiforme* polyphenols have potential as a natural and safe skincare ingredient. These findings provide a scientific basis for developing cosmetic products that are both skin-whitening and anti-aging.

## 1. Introduction

A wealth of structurally diverse bioactive substances with favorable safety profiles, including low cytotoxicity, reproductive toxicity, and genotoxicity, can be found in seaweeds. Concurrently, these compounds exhibit remarkable dermatological benefits, rendering seaweeds promising candidates for functional cosmetic applications [1,2]. Among these constituents, phenolic compounds are abundant, structurally diverse phytochemicals characterized by hydroxylated aromatic frameworks. Biological activities are critically determined by hydroxyl group configuration and abundance, as well as the number of benzene rings [3,4,5]. Seaweed polyphenols exhibit a broad spectrum of dermocosmetic activities, ranging from skin whitening and moisturization to photoprotection, anti-wrinkling, anti-melanogenic, and antioxidant effects [6].

Extrinsic external exposures, such as ultraviolet (UV) radiation, and intrinsic physiological factors both contribute to the complicated biological process of skin aging. Chronic, excessive UV exposure induces a specific condition known as photoaging. Loss of skin suppleness, wrinkles, and the breakdown of collagen and elastin fibers are among its defining characteristics [7,8]. A central issue in skin aging is the upregulation of matrix metalloproteinases (MMPs). The breakdown of collagen and elastin requires these enzymes. Therefore, a fundamental strategy to counteract photoaging is to reduce MMPs levels and stimulate collagen synthesis [9]. Furthermore, because of its special ability to bind water, hyaluronic acid (HA) is an essential component for skin hydration. The aging process is also characterized by reduced HA synthesis, leading to increased skin dryness [10]. Consequently, natural compounds that inhibit MMPs and promote collagen synthesis have become attractive options for the development of anti-aging cosmetics [11,12].

UV radiation is not only a primary inducer of photoaging but also a key external factor stimulating melanin synthesis. Tyrosine is oxidized in multiple steps to yield melanin, a naturally occurring pigment made by melanocytes. Its primary purpose is to shield the genetic material of cells from UV harm [13]. Melanocytes may become dysfunctional due to excessive UV exposure. The accumulation of Reactive Oxygen Species (ROS) during melanogenesis leads to the overactivation of tyrosinase (TYR), may result in hyperpigmentation and even increase the risk of cellular senescence and malignancy [14]. Since TYR is the enzyme that limits the rate at which melanin is synthesized, controlling it has emerged as a key strategy for treating hyperpigmentation problems [15].

Research has demonstrated that seaweed polyphenols exhibit unique advantages in skin protection. These compounds have garnered significant attention for cosmetic applications because of their capacity to alter melanin production pathways and skin photoaging [16]. A notable example is the methanol extract of *Corallina pilulifera*, which has been confirmed to significantly suppress oxidative stress and attenuate UV-induced MMPs expression in human dermal fibroblasts (HDFs), highlighting its promise for developing natural anti-photoaging agents [17]. Phloroglucinol-rich brown algae are another important example. They can prevent melanogenesis by chelating copper ions at the TYR active site [18]. Notably, phlorotannins such as dieckol, dioxinodehydroeckol, and eckol are being intensively investigated as potent TYR inhibitors [19,20,21,22,23,24]. These collective findings point to the therapeutic utility of seaweed polyphenols in treating photoaging and hyperpigmentation.

The economically significant brown seaweed *Sargassum fusiforme* (*S. fusiforme*) is a member of the genus *Sargassum*. It is predominantly distributed in the Northwest Pacific, particularly in China, Japan, and Korea [25]. This seaweed is rich in bioactive nutrients. Research on *S. fusiforme*’s uses in natural medicine, dietary supplements, and cosmetics has advanced significantly lately [26]. According to studies, *S. fusiforme* has a high level of phenolic compounds [27]. *S. fusiforme* polyphenols additionally demonstrate diverse bioactivities, encompassing anti-inflammatory and antioxidant effects [28]. However, the anti-melanogenic potential of SFP remains underexplored in cosmetic applications. Notably, there is a lack of studies using in vivo models, such as zebrafish, which limits the translational relevance of current findings to human skin biology [29]. Additionally, investigations into its anti-photoaging effects are relatively scarce.

The purpose of this study is to use in vitro models to assess the skin-whitening ef-fectiveness of the polyphenol-enriched fraction from *S. fusiforme* (SFP). Furthermore, an in vivo zebrafish model is used to validate SFP’s inhibitory potential against TYR. The study also investigates the effects of SFP on skin-related parameters using a cellular photoaging model. The overall objective is to provide a scientific foundation for developing SFP as a novel natural cosmetic ingredient.

## 2. Materials and Methods

### 2.1. Chemicals and Reagents

TYR, L-tyrosine (L-TYR), elastase, HEPES buffer, Tris buffer, (−)-epigallocatechin gallate (EGCG), N-acetylcysteine (NAC), ninhydrin, and sodium citrate-citric acid buffer were purchased from Shanghai Yuanye Bio-Technology Co., Ltd. (Shanghai, China). L-3,4-dihydroxyphenylalanine (L-DOPA) and Triton X-100 were obtained from Sangon Biotech (Shanghai) Co., Ltd. (Shanghai, China). Collagenase was acquired from Sigma-Aldrich (St. Louis, MO, USA). Fetal bovine serum (FBS), trypsin-EDTA, phosphate-buffered saline (PBS), penicillin-streptomycin solution (P/S), and Dulbecco’s Modified Eagle Medium (DMEM) were supplied by Nanjing BioChannel Biotechnology Co., Ltd (Nanjing, China). For biochemical assays, dimethyl sulfoxide (DMSO) and the human MMP-1 and MMP-3 ELISA kit were obtained from Beijing Solarbio Science & Technology Co., Ltd. (Beijing, China). The BCA protein assay kit and kojic acid (KA) were acquired from Shanghai Acmec Biochemical Technology Co., Ltd (Shanghai, China). The Cell Counting Kit-8 (CCK-8) and ROS assay kit were obtained from Beyotime Biotechnology Co., Ltd. (Shanghai, China). Additionally, ELISA kits for detecting human type I collagen (COL1), elastin, and HA were sourced from Shanghai Jianglai Industrial Co., Ltd. (Shanghai, China). The remaining reagents were all of analytical quality.

### 2.2. Preparation and Chemical Characterization of SFP

The seaweed *S. fusiforme* was collected from Dongtou Island, Zhejiang Province, China. The collected samples were subsequently ground into a fine powder following air-drying, then preserved at 4 °C prior to extraction. A slightly modified version of a previously published method was used to extract and purify polyphenols [30]. Briefly, 26% (*v*/*v*) ethanol was used to extract the seaweed powder at 70 °C for 40 min with a 1:40 (*w*/*v*) solid-to-liquid proportion. XDA-7 macroporous adsorption resin was then used to purify the resultant crude extract. Rotary evaporation was used to concentrate the eluate after the target fraction was eluted. The concentrate was finally lyophilized to obtain SFP powder. Total phenolics in the SFP extract were quantified using the Folin–Ciocalteu reagent with a microplate reader (Tecan, Morrisville, NC, USA). Gallic acid equivalents (GAEs) in milligrams per gram of sample dry weight were used to express the results.

The chemical composition of SFP was characterized by LC-MS/MS analysis, which was commissioned to Qingdao Standard Testing Co., Ltd. (Qingdao, China). A Vanquish UHPLC system connected to a Q Exactive HF high-resolution mass spectrometer (Thermo Fisher Scientific, Waltham, MA, USA) was used for the analyses. Chromatographic separation was performed on a Zorbax Eclipse C18 column with a binary gradient of 0.1% formic acid in water (A) and acetonitrile (B). Detection was carried out in both positive- and negative-ion modes using full-scan MS (m/z 100–1500, resolution 120,000) followed by data-dependent MS/MS (resolution 6000, HCD fragmentation).

### 2.3. In Vitro Collagenase and Elastase Inhibition Assays

SFP inhibition of Collagenase and Elastase was assessed according to an established protocol [31]. The positive control, EGCG, was used at 114 μg/mL.

### 2.4. In Vitro TYR Inhibition Assay

With a few minor adjustments, TYR activity was measured as previously described [32]. Briefly, L-TYR and L-DOPA were used as substrates to assess TYR activity. The reaction mixture consisted of the substrate, TYR solution (500 U/mL), PBS buffer, and various concentrations of SFP. A microplate reader captured absorbance readings at 475 nm right after 5-min incubation at 37 °C. KA served as a positive control. The concentration of TYR was changed while the concentration of L-DOPA was fixed in order to examine the reversibility of SFP-induced TYR inhibition. Reaction rates were measured at different SFP concentrations (recorded at 475 nm every 30 s for 5 min). Plots of reaction rate versus enzyme concentration were constructed to determine whether inhibition was reversible, based on the linearity of the data passing through the origin. Enzyme kinetics assays were performed to determine the inhibition mode of SFP against TYR. With a fixed enzyme concentration and varying L-DOPA concentrations, reaction rates were measured at different SFP concentrations. The inhibition mode and inhibition constant (*Ki*) were analyzed using Lineweaver-Burk plots.

### 2.5. Cell Culture and Cell Viability

A375 human melanoma cells (Cat. No. FH0178) were purchased from Shanghai Fuheng Biotechnology Co., Ltd. (Shanghai, China). HDF cells (Cat. No. PC2031) were obtained from Guangdong BioCell Biotechnology Co., Ltd. (Dongguan, China). High-glucose DMEM supplemented with 15% FBS and 1% P/S was used to cultivate A375 cells. Low-glucose DMEM with 10% FBS was used to sustain HDF cells, with daily medium changes. After all cells were grown at 37 °C in a humidified atmosphere with 5% CO_2_, cytotoxicity was measured using the CCK-8 assay. A375 and HDF cells were plated at 1 × 10^4^ and 7 × 10^3^ cells/well, respectively, in a 96-well format. Cells were treated with graded doses of SFP for a full day. After adding the CCK-8 solution, the cells were cultured for an additional 2 h to determine their viability. Absorbance at 450 nm was read on a microplate reader.

### 2.6. Determination of Intracellular Melanin Content and TYR Activity in A375 Cells

In 12-well plates, A375 cells were planted at a density of 1.0 × 10^3^ cells/well, and they were incubated for a whole night. Based on cytotoxicity results, we exposed cells to SFP at concentrations ranging from 25 to 150 μg/mL for 48 h. Following PBS wash, a solution of 1 M NaOH with 10% DMSO was used to resuspend and dissolve the cell pellets. The mixture was heated to 95 °C for 1 h while being vortexed every 10 min. After cooling, absorbance at 405 nm was used to calculate the melanin content. TYR activity was measured using a slightly modified version of the previously published method [33]. A375 cells were plated in 6-well plates at 4.0 × 10^5^ cells/well. Subsequently, they were treated with SFP (25–150 μg/mL) for 60 h. Following PBS wash, Cells were lysed in 1% Triton X-100 after collection. After freeze-thaw (−80 °C, 30 min), lysates were centrifuged. The supernatant was collected as crude TYR extract. The BCA assay quantified protein concentrations, which were then normalized to ensure uniformity across all samples. Absorbance was then measured at 475 nm after incubating the extract with the L-DOPA solution for 2 h at 37 °C. KA (1.5 mg/mL) was used as the positive control for both assays.

### 2.7. Measurement of Intracellular ROS Levels in A375 Cells

The fluorescent probe 2′,7′-dichlorodihydrofluorescein diacetate (DCFH-DA) was used to measure intracellular reactive ROS levels. Black 96-well plates were used to seed A375 cells at a density of 1 × 10^4^ cells/well. After adherence, cells were pretreated with SFP at 50, 100, 150 μg/mL for 12 h. They were subjected to H_2_O_2_ (600 μM) for 6 h in order to cause oxidative stress. Cells were stained with 10 μM DCFH-DA (20 min, 37 °C, dark). Excess probe was removed by washing with fresh culture medium. A microplate reader (BioTek, Winooski, VT, USA) with excitation and emission wavelengths set to 485 nm and 530 nm, respectively, was used to measure fluorescence intensity.

### 2.8. Zebrafish Embryo Culture and Toxicity Assessment

Weifang Jinghe Biotechnology Co., Ltd. (Weifang, China) supplied the wild-type zebrafish (AB strain). The fish were housed in a controlled setting with a 14/10-h light/dark cycle and three daily meals at 27 ± 0.5 °C. Adult zebrafish were randomly paired in separate tanks in a 1:1 male-to-female ratio the day prior to the studies. Spawning was triggered by light onset the following morning. Embryos were collected, rinsed with system water to remove debris, and maintained in standard embryo medium. They were then incubated at 28 ± 0.5 °C for subsequent use. Normally formed embryos at 6–8 h post-fertilization (hpf) were chosen at random and put into 6-well plates with 30 embryos per well for the toxicity assessment. The group size (n = 30) was determined empirically based on preliminary data and standard practice in this field. The SFP concentrations to which the embryos were exposed ranged from 5 to 200 μg/mL. KA at 50 μg/mL functioned as the positive control. The exposure continued until 5 days post-fertilization (dpf). At this endpoint, embryo mortality, hatching rate, and incidence of morphological malformations were recorded and calculated.

### 2.9. Melanogenesis on the Body Surface of Zebrafish Embryos

Zebrafish embryos were treated with SFP (5–20 μg/mL) and KA (50 μg/mL, positive control) until 3 dpf. Following exposure, embryos were anesthetized with tricaine methanesulfonate (MS-222, obtained from Weifang Jinghe Biotechnology Co., Ltd., Weifang, China) and imaged using a stereo microscope (Nikon, Tokyo, Japan). The distribution of melanin granules on the body surface was documented, and the melanin content was subsequently quantified using ImageJ 1.54g (National Institutes of Health, Bethesda, MD, USA). For each treatment group, the relative melanin content was calculated based on this analysis.

### 2.10. Determination of Melanin Content and TYR Activity in Zebrafish

A slightly modified version of the previously reported method was used to measure the melanin content of zebrafish [34]. Briefly, after washing three times with PBS, embryos at 3 dpf were homogenized using an ultrasonic cell disruptor (HUXI, Shanghai, China). The pellet was resuspended in 600 μL of 1 M NaOH with 10% DMSO following centrifugation of the homogenate. For 1 h, the mixture was heated to 80 °C while being vortexed every 20 min. Post-cooling, absorbance values were acquired at a wavelength of 405 nm. Embryos at 3 dpf were used to measure TYR activity. Following a PBS wash, embryos were lysed in PBS containing 1% Triton X-100, then sonicated for 10 s. The crude TYR extract was obtained by centrifuging the lysate and collecting the supernatant. The BCA assay quantified protein concentration, and the values were normalized to equalize concentrations across samples. Subsequently, we mixed the extract with L-DOPA and incubated the mixture for 2 h at 37 °C. At 475 nm, absorbance was measured. For both tests, KA (50 μg/mL) served as the positive control.

### 2.11. Ultraviolet A (UVA) Radiation Dose Selection and Photoprotective Effect of SFP

HDFs were seeded in 96-well plates. After washing with PBS, HDFs were exposed to UVA doses ranging from 10 to 640 mJ/cm^2^ using a TL 40 W/10 UVA lamp (Philips, Eindhoven, The Netherlands; 340–400 nm, peak at 369 nm, irradiance of 10.78 mW/cm^2^). The blank control was shielded with aluminum foil. Following irradiation, cells were cultured in fresh medium for 24 h. The CCK-8 assay was used to assess cell viability and establish the optimal dose for further studies. Photoprotective Effect Evaluation: HDF cells were pretreated with SFP (15, 30, and 60 μg/mL) or TGF-β1 (100 ng/mL, positive control) for 12 h. The normal control group (no UVA, no treatment) and the UVA model group (UVA only) received the vehicle. All groups except controls received 80 mJ/cm^2^ of UVA radiation. After irradiation, cells were cultured for an additional 24 h before CCK-8 viability assessment.

### 2.12. Determination of Skin-Related Parameters Under UV Irradiation

HDF cells were seeded at 1.5 × 10^3^ cells/well in 6-well plates and subsequently treated with SFP (15, 30, and 60 μg/mL) and positive controls (100 ng/mL TGF-β1 and 1 mM NAC) for 24 h. Subsequently, following washing with PBS, except for the normal control group, the cells were exposed to 80 mJ/cm^2^ UVA, which was shielded from irradiation. Following irradiation, the cells were cultured for an additional 24 h. ELISA kits were used to measure the amounts of COL1, elastin, HA, MMP-1, and MMP-3 in culture supernatants.

### 2.13. Statistical Analysis

Every experiment was carried out three times. The mean ± standard deviation (SD) is used to express data. GraphPad Prism 8 (GraphPad Software, San Diego, CA, USA) was used for statistical analyses. A one-way ANOVA and Duncan’s multiple-comparison test were used to compare different groups. *p* < 0.05 was considered statistically significant.

## 3. Results

### 3.1. Chemical Characterization of SFP

Polyphenols were extracted from *S. fusiforme* using a 26% aqueous ethanol solution through two extraction cycles, achieving a polyphenol extraction rate of 10.672 mg GAE/g dry weight. A crude polyphenol powder was obtained by lyophilizing the mixed extracts after they were concentrated by rotary evaporation under low pressure. This crude powder was purified using XDA-7 macroporous adsorption resin. The target polyphenol fraction was eluted, concentrated, and lyophilized, yielding the final purified SFP powder. The initial crude extract contained 1.5% (*w*/*w*) polyphenols. After resin purification, the polyphenol content increased to over 33.1% (*w*/*w*), a more than 20-fold increase. For every experiment that followed, the purified SFP was kept at −20 °C.

Polyphenolic compounds in the ethanol extract of *S. fusiforme* were analyzed by LC–MS/MS. MS/MS fragmentation patterns were interpreted based on accurate mass, retention time, and characteristic fragment ions. Structural annotation was performed by comparison with literature data and spectral libraries. A total of six phenolic compounds were tentatively identified, belonging to four major classes: phlorotannins, phenolics, flavonoids, and cinnamic acid derivatives. Among them, the major components were isoquercitrin (158.95 μg/g, flavonoid) and caffeic acid (154.78 μg/g, cinnamic acid derivative). Other representative compounds included 6-gingerol (24.93 μg/g, phenolic), ferulaldehyde (1.24 μg/g, phenolic), and fucodiphlorethol G (0.40 μg/g, phlorotannin). These results indicate that SFP is a complex polyphenolic mixture dominated by flavonoids and cinnamic acid derivatives, rather than a purified single compound or a pure phenolic mixture.

### 3.2. Inhibitory Effects of SFP on Collagenase and Elastase

**Figure 1:**
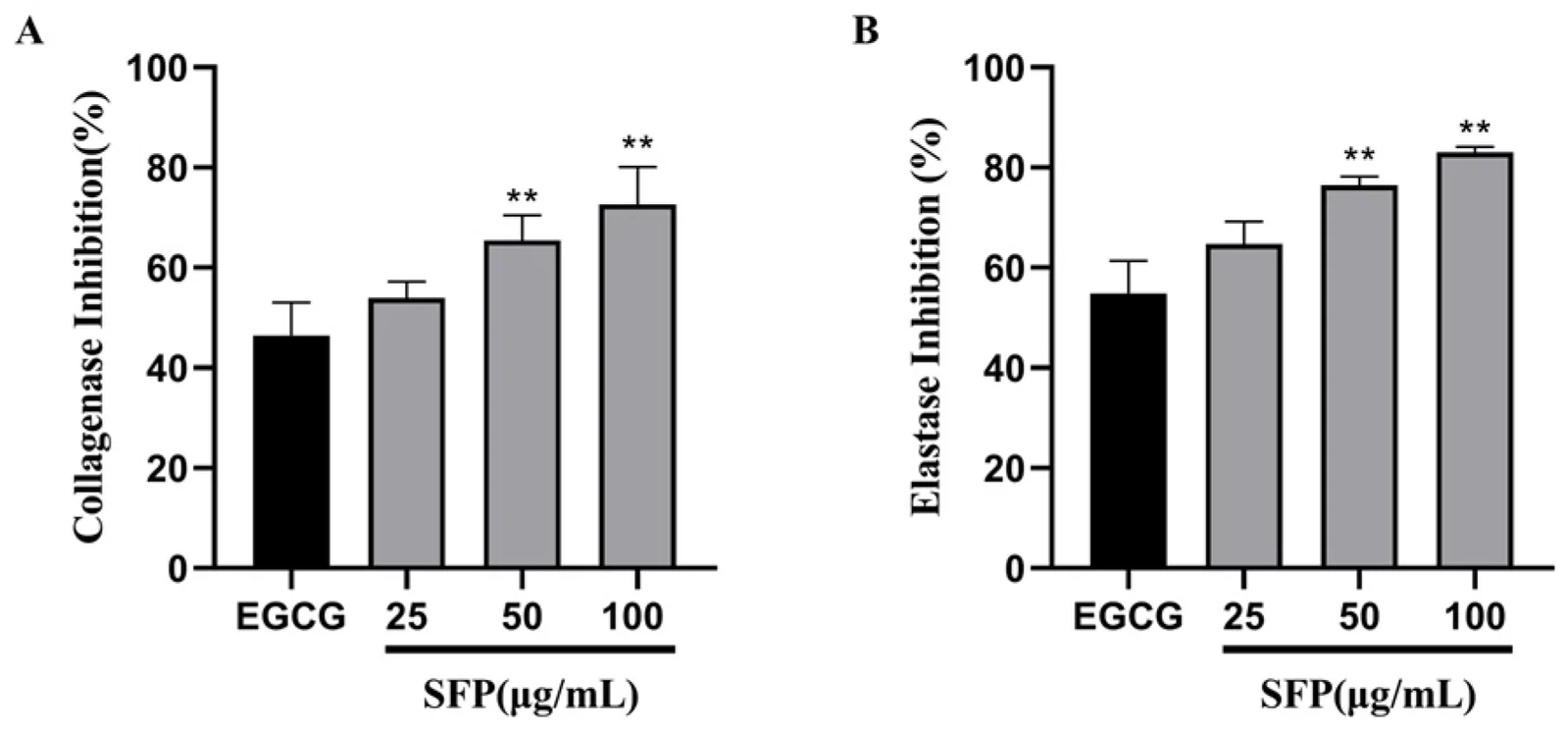
Inhibitory effects of SFP on collagenase (**A**) and elastase (**B**). Values are expressed as mean ± SD (*n* = 3). Values differed significantly from those of the EGCG group (** *p* < 0.01).

Collagen and elastin, two crucial matrix proteins in the extracellular matrix (ECM), are broken down by the enzymes collagenase and elastase. This degradation process is one of the primary causes of skin aging. This study assessed the inhibitory effects of SFP on these enzymes, using EGCG as a positive control at a concentration of 114 μg/mL. SFP exhibited dose-dependent inhibition of both collagenase and elastase that was stronger than that of EGCG (Figure 1). Collagenase inhibition by SFP reached 65.45% and 72.61% at 50 and 100 μg/mL, respectively. These inhibition rates were significantly higher than the 46.62% inhibition observed for EGCG (Figure 1A). SFP at the same concentrations also showed substantial inhibitory activity against elastase (Figure 1B). These findings demonstrate that SFP effectively inhibits collagenase and elastase in vitro. This activity suggests that SFP has the potential to protect skin ECM from degradation, thereby alleviating signs of skin aging.

### 3.3. Inhibitory Effect of SFP on TYR Activity

#### 3.3.1. Inhibition of TYR by SFP

**Figure 2:**
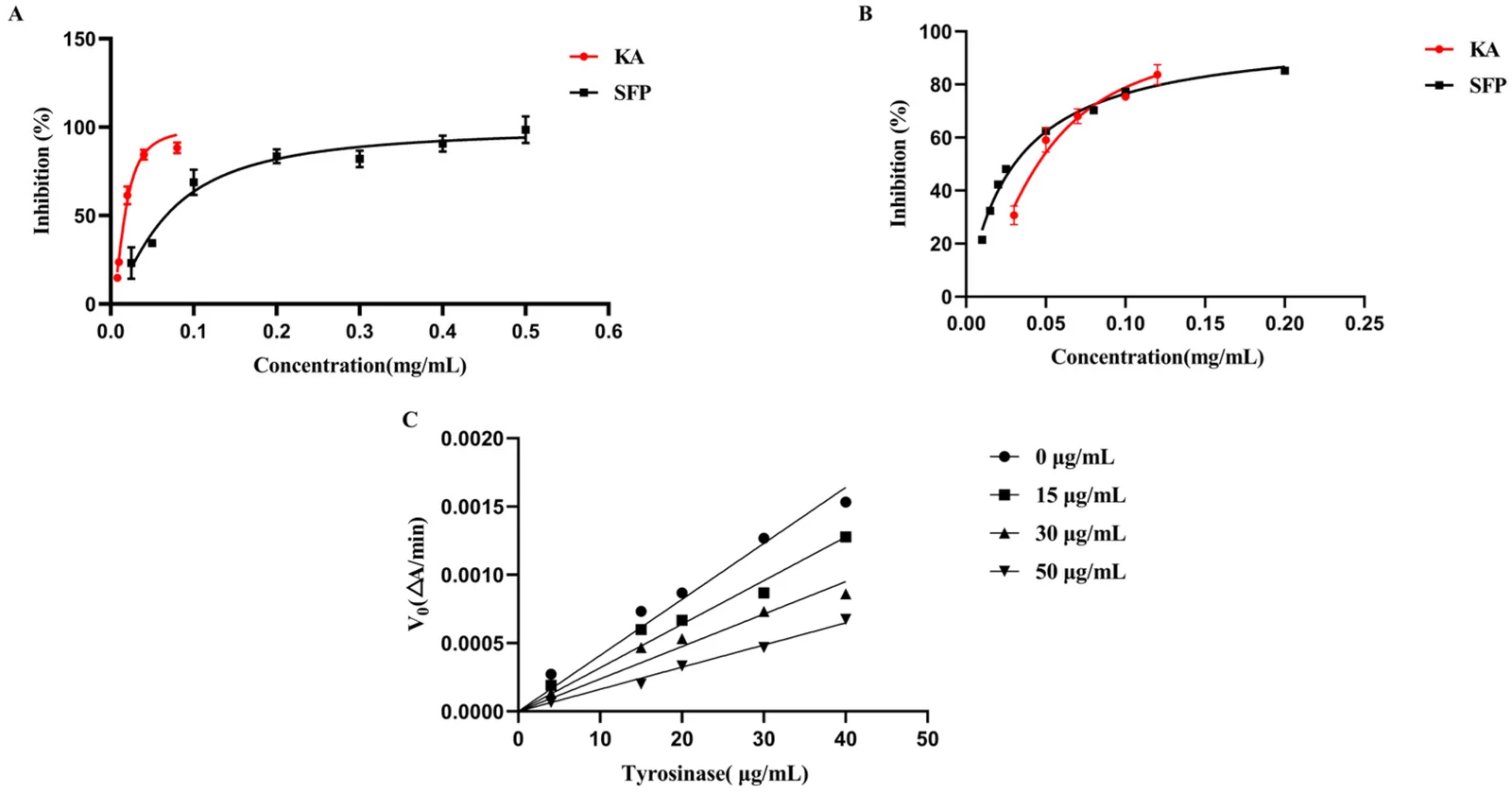
Inhibitory effects and mechanism of SFP on tyrosinase (TYR) activities. Concentration-dependent graph of TYR inhibitory activity of SFP along with standard kojic acid (KA) using L-tyrosine (L-TYR) (**A**) and L-3,4-dihydroxyphenylalanine (L-DOPA) (**B**) as substrates. Reversible inhibition of TYR by SFP (**C**). Values are expressed as mean ± SD (*n* = 3).

As a monophenolase and diphenolase, TYR demonstrates catalytic activity. L-TYR and L-DOPA were used as specific substrates to assess SFP’s inhibitory effects on them, respectively. In a concentration-dependent way, SFP suppressed TYR’s monophenolase and diphenolase activities (Figure 2A,B). We subsequently determined IC_50_ values. SFP exhibited IC_50_ values of 0.067 mg/mL (monophenolase) and 0.03 mg/mL (diphenolase), while KA, a standard TYR inhibitor, showed 0.017 mg/mL and 0.045 mg/mL, respectively. These findings demonstrate that SFP potently inhibits TYR, exhibiting approximately 1.5-fold greater potency than KA against diphenolase activity (lower IC_50_ values indicating greater potency). These findings establish that SFP effectively inhibits TYR activity.

#### 3.3.2. Inhibitory Mechanism of SFP on TYR

To determine the reversibility of inhibition, assays were conducted with a fixed L-DOPA concentration, varying TYR amounts, and different SFP concentrations. As shown in Figure 2C, plots of reaction rate versus enzyme concentration yielded straight lines for each SFP concentration. As SFP concentrations increased, the slopes of these lines dropped, suggesting decreasing TYR activity. All lines passed through the origin, demonstrating that SFP is a reversible inhibitor of TYR. The reversibility of this inhibition is attributed to non-covalent interactions between SFP and the enzyme, resulting in a reduced oxidation rate of the diphenolase substrate L-DOPA. Following inhibitor removal, TYR can restore its original catalytic activity by dissociating these non-covalent bonds.

#### 3.3.3. Inhibition Type of SFP on TYR

**Figure 3:**
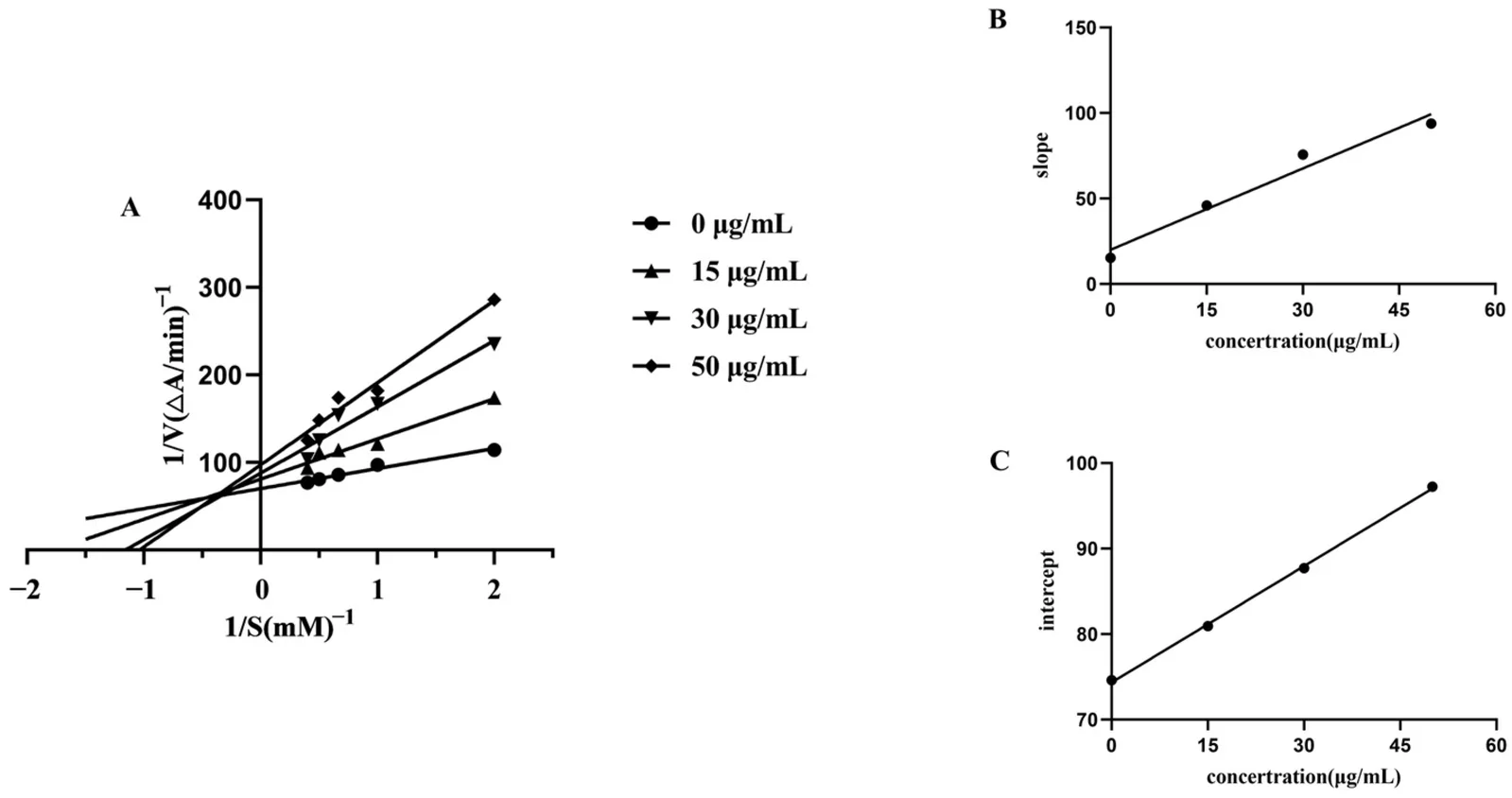
Inhibition type of SFP on TYR. SFP inhibits TYR activity as shown by Lineweaver-Burk plots using L-DOPA as substrate (**A**). Secondary plots of slopes (**B**) and intercepts (**C**) versus SFP concentration for determination of inhibition constants.

Lineweaver-Burk plots revealed the manner of SFP inhibition on TYR (Figure 3A). As the SFP concentration increased, the x-intercept decreased, and the y-intercept increased. All lines intersected in the second quadrant. This kinetic pattern is characteristic of mixed-type inhibition. SFP exhibits affinity for the free enzyme in addition to the enzyme-substrate complex. The inhibition constants were derived from secondary plots. We plotted the slope (Figure 3B) and y-intercept (Figure 3C) from the double-reciprocal plots against SFP concentration. From these plots, *Ki* for binding to the free enzyme was calculated to be 12.64 μg/mL. The dissociation constant (*Kis*) for binding to the enzyme-substrate complex was 164 μg/mL. *Ki* < *Kis* implies a stronger affinity of SFP for the free enzyme compared with the substrate-bound complex. A lower *Ki* value directly correlates with stronger enzyme-inhibitor binding and higher inhibitory potency. These kinetic data confirm that SFP acts as a potent, mixed-type, reversible inhibitor of TYR. This mechanism aligns with findings from related studies, supporting the role of SFP as an effective TYR inhibitor that does not permanently inactivate the enzyme.

### 3.4. Cytotoxic Effects of SFP on A375 and HDF Cells

**Figure 4:**
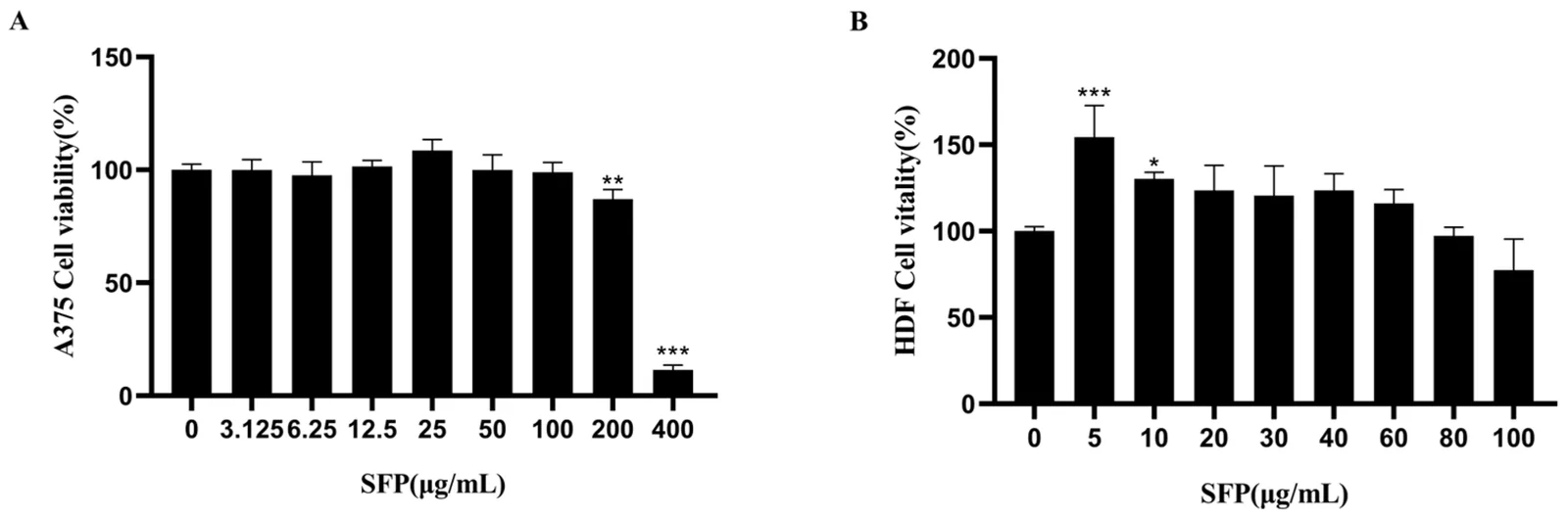
Effect of SFP on A375 (**A**) and HDF (**B**) cell viability. Values are expressed as mean ± SD (*n* = 3). * *p* < 0.05, ** *p* < 0.01, and *** *p* < 0.001 in comparison to the control group indicate statistical significance.

The cytotoxicity of SFP was assessed to determine safe working concentrations. The CCK-8 assay assessed cell viability. For A375 cells (Figure 4A), SFP at 3.125–100 μg/mL did not significantly reduce cell viability. Significant cytotoxicity (*p* < 0.01) was observed only at 200 μg/mL. Therefore, SFP concentrations below 200 μg/mL were selected for use in all subsequent experiments. Exposure to SFP at concentrations up to 80 μg/mL for 24 h did not result in substantial cytotoxicity for HDF cells (Figure 4B). Notably, concentrations below 60 μg/mL showed a mild, non-toxic proliferative effect compared to the untreated control. These findings led to the selection of SFP doses of 15, 30, and 60 μg/mL for further cellular studies. These doses are within the non-toxic range for both cell lines.

### 3.5. Effects of SFP on Melanin Content and TYR Activity in A375 Cells

**Figure 5:**
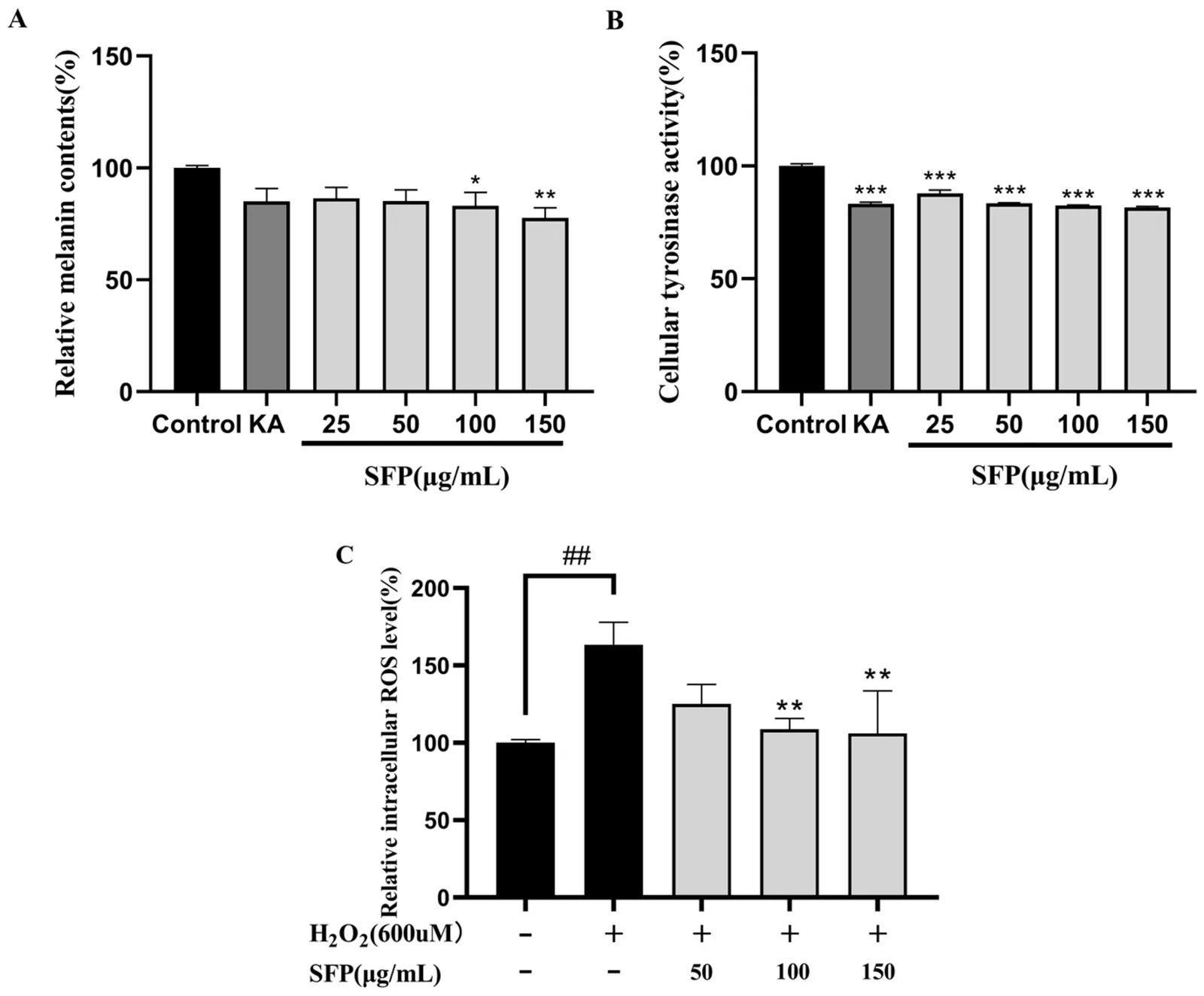
Anti-melanogenic activity of SFP and its influence on reactive oxygen species (ROS) levels. (**A**) The impact of SFP on melanin content in A375 cells. * *p* < 0.05 and ** *p* < 0.01 indicate significant differences versus the control group. (**B**) The inhibitory effect of SFP on TYR activity in A375 cells. *** *p* < 0.001 indicates significant difference compared with the control group. (**C**) The impact of different SFP concentrations on ROS levels in A375 cells treated with H_2_O_2_. ^##^ *p* < 0.01 indicates significant changes from the blank group, and ** *p* < 0.01 indicates significant differences from the H_2_O_2_-induced injury model group. Values are expressed as mean ± SD (*n* = 3).

We assessed SFP’s impact on TYR activity and melanin production in A375 cells. SFP treatment significantly inhibited melanin production in a dose-dependent manner (Figure 5A). Compared to the control, melanin content was significantly reduced at 100 μg/mL (*p* < 0.05) and 150 μg/mL (*p* < 0.01). At 150 μg/mL, melanin content decreased by 22.27%. Likewise, intracellular TYR activity was dose-dependently suppressed by SFP (Figure 5B). The inhibitory potency of SFP was comparable to that of KA, despite being applied at a concentration over ten times lower. These findings indicate that SFP effectively reduces melanin synthesis in A375 cells, mediated through potent inhibition of intracellular TYR activity.

### 3.6. Effect of SFP on Intracellular ROS in A375 Cells

Oxidative stress, driven by excessive ROS, plays a key role in melanogenesis. Therefore, reducing ROS levels is a valid strategy to counteract pigmentation. We investigated whether SFP could reduce oxidative stress by measuring intracellular ROS in A375 cells exposed to 600 μM H_2_O_2_ (Figure 5C). H_2_O_2_ treatment alone significantly elevated ROS levels compared to the control, confirming the induction of oxidative stress. Pretreatment with SFP effectively and concentration-dependently suppressed this H_2_O_2_-induced increase in ROS. Significant inhibition (*p* < 0.01) was observed at 100, 150 μg/mL SFP compared to the H_2_O_2_-injury model group. These findings confirm that SFP is a polyphenol-rich extract with potent antioxidant activity. It can mitigate intracellular oxidative stress, a mechanism contributing to its observed anti-pigmentation effect.

### 3.7. Effects of SFP on Zebrafish Embryo Toxicity and Relative Body Surface Melanin Content

**Figure 6:**
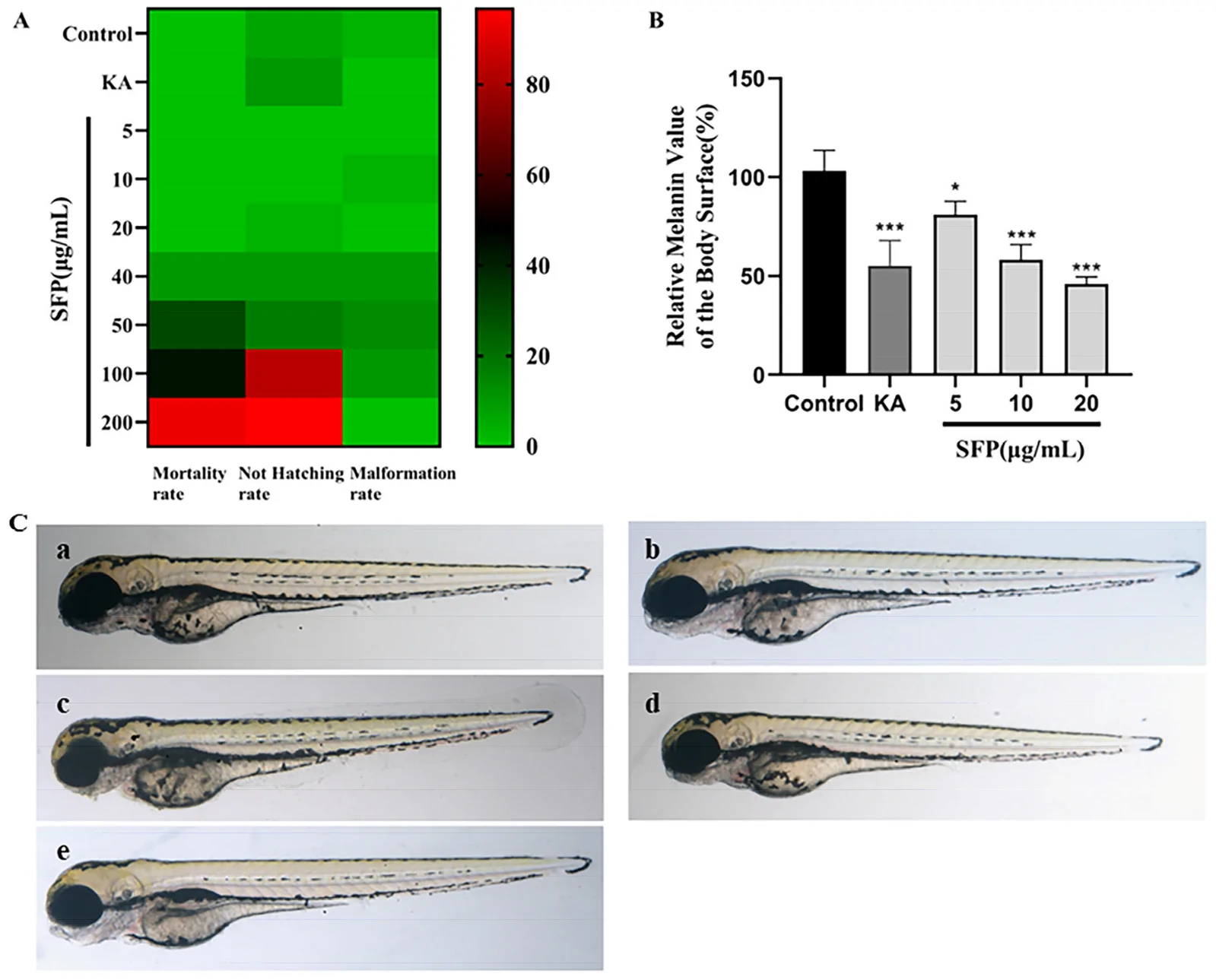
Effects of SFP on embryo toxicity and body surface melanin content in zebrafish. (**A**) Heatmap analysis of SFP effects on zebrafish embryo mortality, not hatching, and malformation. Zebrafish larvae were exposed to SFP for 72 h, with KA serving as a positive control. (**B**) The effect of SFP on relative melanin content on the body surface of zebrafish embryos. Values are expressed as mean ± SD (*n* = 6). Significant differences from the control are denoted as * *p* < 0.05 and *** *p* < 0.001. (**C**) Morphological images of zebrafish. Panels a to e depict the effects observed in the Control group (**a**), KA group (**b**), and 5 (**c**), 10 (**d**), and 20 (**e**) μg/mL SFP treatment groups on body surface melanin formation, respectively.

A toxicity assessment was conducted in zebrafish embryos to determine a safe concentration range for SFP (Figure 6A). SFP concentrations above 40 μg/mL significantly increased embryo mortality, reduced hatching rates, and elevated malformation rates. At 200 μg/mL, most embryos died before hatching. Conversely, dosages up to 20 μg/mL had no discernible effect on hatching, deformities, or mortality when compared to the control. The positive control, KA, was also found to be non-toxic. Therefore, SFP at 5, 10, and 20 μg/mL were selected for all subsequent zebrafish experiments. The effect of SFP on melanin accumulation is illustrated in Figure 6C. SFP treatment visibly and concentration-dependently reduced melanin pigmentation on the embryo surface, particularly in the yolk sac and ventral stripe regions. Higher concentrations of SFP resulted in a lighter body color. The relative melanin content was quantified (Figure 6B). Treatment with 10 μg/mL SFP resulted in 58.2% relative melanin content. At its tested concentration, KA reduced melanin to 55.05%. This result demonstrates that SFP exerts a stronger inhibitory effect on in vivo melanogenesis than the standard inhibitor KA.

### 3.8. Effects of SFP on Melanin Synthesis and TYR Activity in Zebrafish Embryos

**Figure 7:**
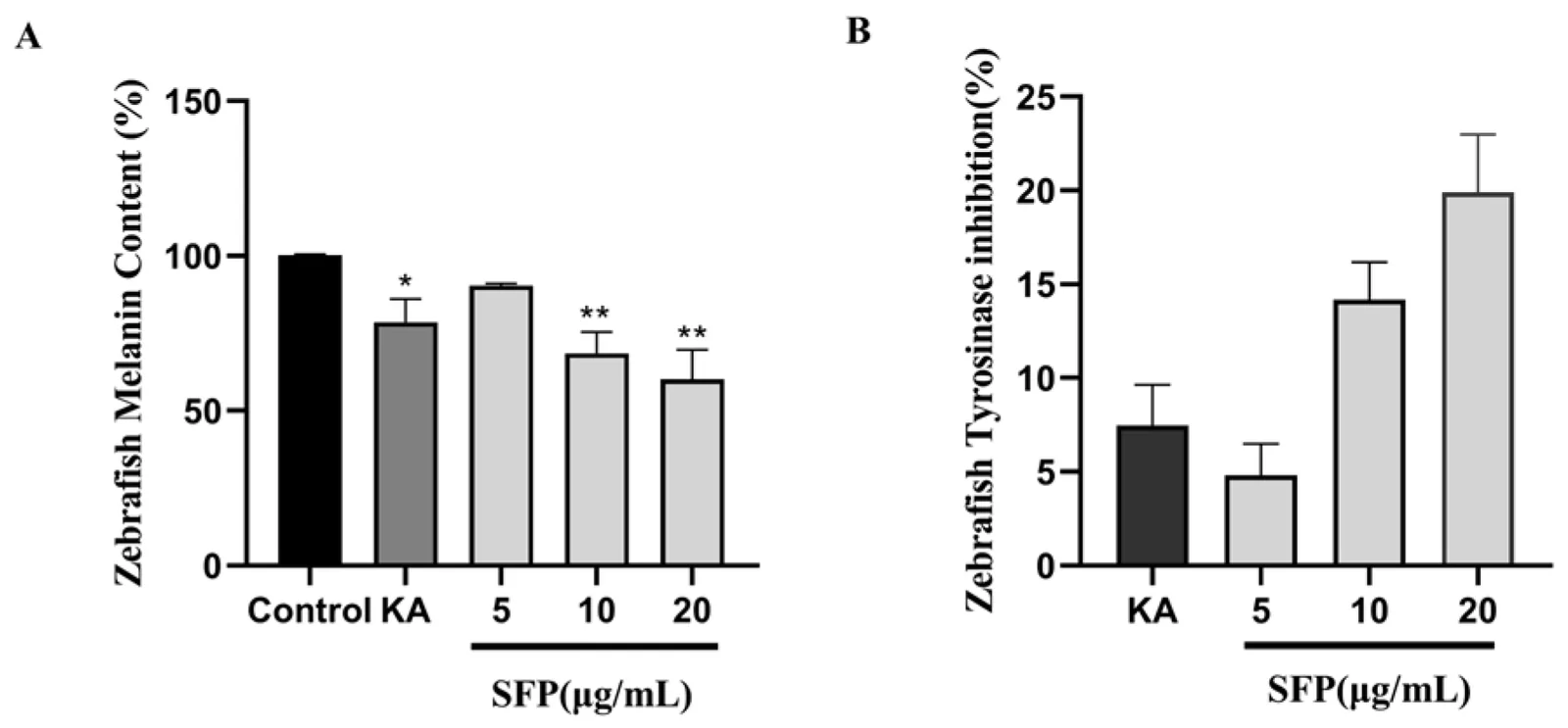
Anti-melanogenic activity of SFP in zebrafish embryos. (**A**) SFP’s impact on zebrafish melanin content in vivo (**B**) Inhibitory effect of SFP on TYR activity in zebrafish embryos. Values are expressed as mean ± SD (*n* = 3). Significance relative to control: * *p* < 0.05, ** *p* < 0.01.

Using zebrafish embryos, we evaluated SFP’s anti-melanogenic efficacy in vivo (Figure 7). Embryos were administered SFP at 5–20 μg/mL for 72 h. Treatment with 10 and 20 μg/mL SFP significantly reduced melanin content to 68.53% and 60.17% of the control level, respectively (Figure 7A). At 10 μg/mL, SFP exhibited a stronger inhibitory effect than the positive control, KA. This observation was consistent with representative images of the embryos. It demonstrates potent anti-melanogenic effects in vivo, matching the body surface results. Additionally, SFP showed concentration-dependent inhibition of TYR activity in embryo extracts (Figure 7B). These results suggest that SFP effectively attenuates melanogenesis by inhibiting TYR activity in zebrafish.

### 3.9. Optimal UVA Dose and Photoprotective Effect of SFP on HDF Cells

**Figure 8:**
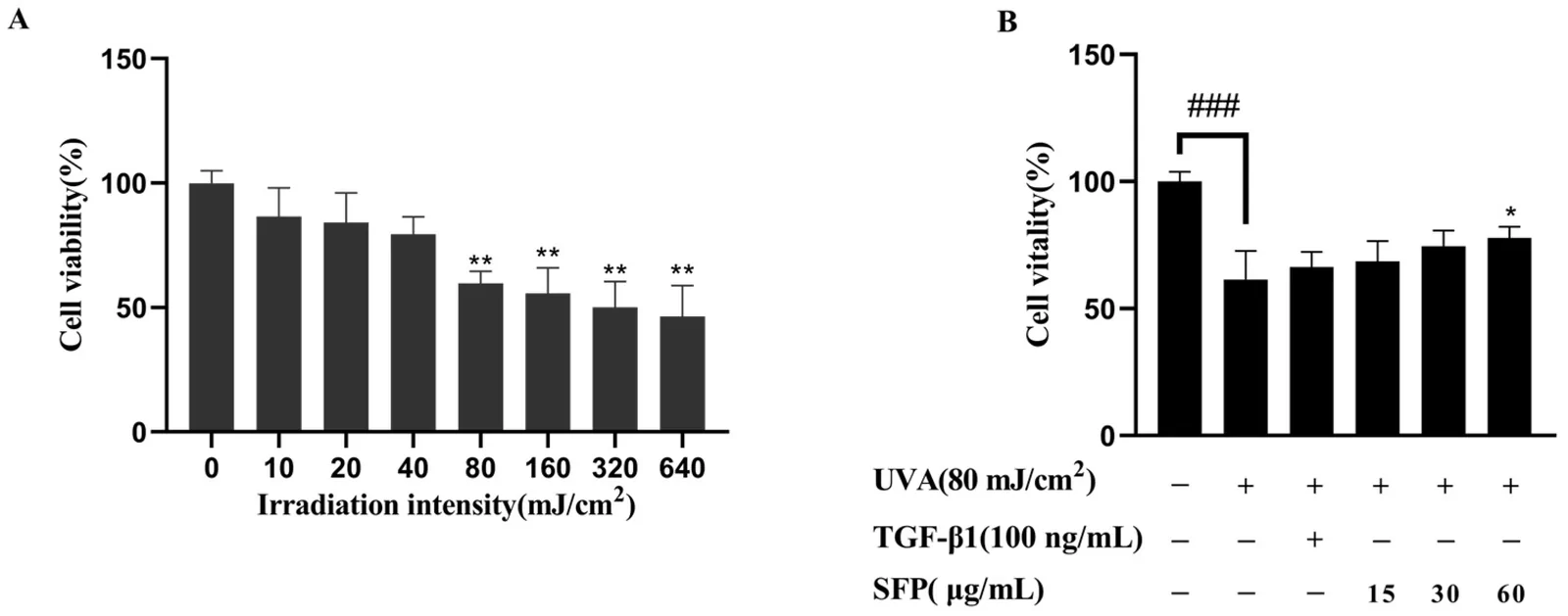
Photoprotective effect of SFP on HDF Cells. (**A**) The impact of varying UVA irradiation intensities on HDF cells. ** *p* < 0.01 indicate significant differences from the control group. (**B**) The protective effect of SFP against UVA-induced photoaging in HDFs. Values are presented as mean ± SD (*n* = 3). ^###^ *p* < 0.001 indicate significant differences from the control group. * *p* < 0.05 indicate significant differences compared to the UVA-only group.

HDF cells were exposed to UVA at doses from 10 to 640 mJ/cm^2^. After 24 h of radiation, cell viability was assessed (Figure 8A). Cell viability decreased in a dose-dependent manner when exposed to UVA radiation. Compared with the non-irradiated control, radiation at 80 mJ/cm^2^ or higher significantly reduced viability (*p* < 0.01). A dose of 80 mJ/cm^2^ was selected for subsequent experiments. This dose reduced cell viability by approximately 40% while maintaining acceptable overall cell morphology. The ability of SFP to protect against UVA damage was then evaluated (Figure 8B). Treatment with 80 mJ/cm^2^ UVA significantly lowered HDF cell viability compared to the control, indicating that UVA irradiation caused damage to HDF cells. Notably, pretreatment with 60 µg/mL SFP significantly improved cell viability compared to the UVA-only group (*p* < 0.05), showing a concentration-related protective trend. These results suggest that SFP possess photoprotective potential against UVA-induced injury in HDF cells.

### 3.10. Determination of Skin-Related Parameters in UVA-Irradiated HDF Cells

#### 3.10.1. Levels of Collagen and Elastin

**Figure 9:**
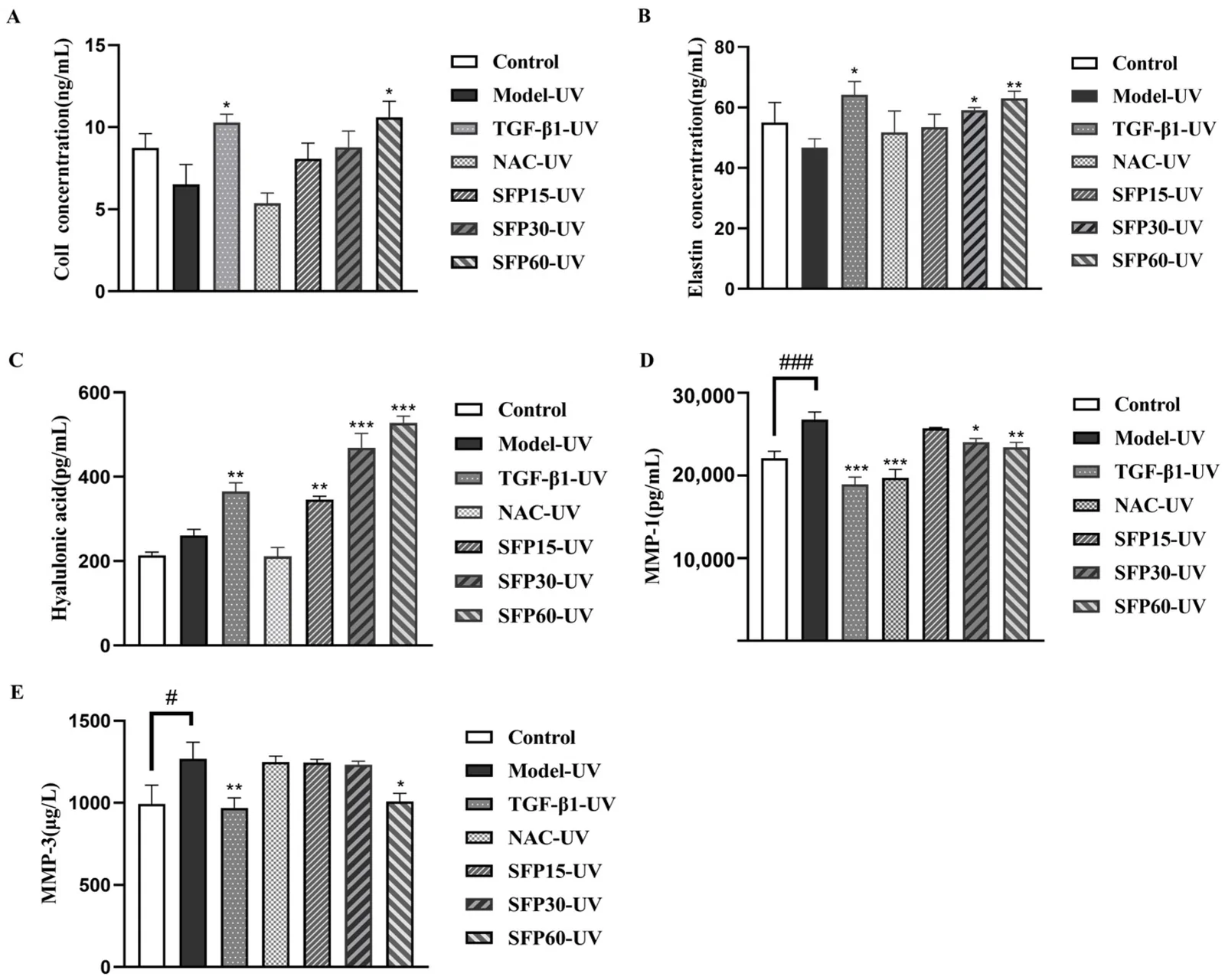
SFP effects on skin-related parameters in UVA-irradiated HDF cells. Type I collagen (COL1) levels (**A**); elastin levels (**B**); hyaluronic acid (HA) levels (**C**); matrix metalloproteinase-1 (MMP-1) levels (**D**); and matrix metalloproteinase-3 (MMP-3) levels (**E**) in HDF cells after UVA irradiation. Values are presented as mean ± SD (*n* = 3). * *p* < 0.05, ** *p* < 0.01, *** *p* < 0.001 indicate significant differences compared to the UVA-induced injury model group; ^#^ *p* < 0.05, ^###^ *p* < 0.001 indicate significant differences compared to the control group.

In this study, COL1 and elastin expression levels were reduced by UVA irradiation alone compared with the control group. However, these differences were not statistically significant. Notably, the 60 μg/mL SFP group (SFP60-UV) significantly increased COL1 levels, with an effect comparable to that of the TGF-β1 positive control (Figure 9A). For elastin, the 30 μg/mL SFP group (SFP30-UV) and SFP60-UV groups significantly enhanced elastin levels compared with the UVA-irradiated model group (Model-UV) (Figure 9B). The restorative effects of all SFP concentrations on elastin were weaker than those of TGF-β1 but stronger than those of NAC. These results demonstrate that SFP restored COL1 and elastin levels to some extent in UVA-irradiated HDF cells, with a concentration-dependent trend.

#### 3.10.2. Hyaluronic Acid Levels

The HA level in the Model-UV group was slightly higher than that in the control group, suggesting that UVA irradiation may have induced a cellular stress response. However, this difference was not statistically significant. SFP treatment dramatically increased HA levels in a concentration-dependent manner compared with the Model-UV group (Figure 9C). The TGF-β1 positive control had lower HA levels than the SFP30-UV and SFP60-UV groups. In contrast, NAC showed no significant effect on HA levels. COL1, elastin, and HA are key components of the dermal ECM and are commonly used as indicators for evaluating the anti-wrinkle efficacy of cosmetic ingredients. Collectively, these results suggest that SFP, at higher concentrations, exerts a partial ameliorative effect on ECM-related parameters (COL1, elastin, and HA) in UVA-irradiated HDF cells, implying a potential anti-wrinkle activity.

#### 3.10.3. Matrix Metalloproteinase Levels

We investigated how SFP reduced the expression of MMP-1 and MMP-3 generated by UVA. UVA irradiation at 80 mJ/cm^2^ led to a marked increase in MMP-1 and MMP-3 levels in HDF cells. Various concentrations of SFP effectively reduced the UVA-induced elevation in MMP-1 levels. The SFP60-UV group showed the greatest inhibitory impact. All SFP concentrations were more effective at reducing MMP-1 levels than both the positive controls (TGF-β1 and NAC). For MMP-3 (Figure 9E), the 15 and 30 μg/mL SFP groups (SFP15-UV) did not differ significantly from the Model-UV group. However, the SFP60-UV group significantly reduced MMP-3 levels, though this reduction was less pronounced than that achieved by TGF-β1. The NAC group showed no significant effect on MMP-3 levels. In conclusion, SFP prevents UVA-induced upregulation of MMP-1 and MMP-3 in HDF cells. This activity mitigates cellular damage and counteracts photoaging.

## 4. Discussion

Melanin is synthesized and accumulated within melanosomes. After being transferred to keratinocytes, melanin is distributed and ultimately metabolized, processes that determine skin pigmentation [35,36]. The primary enzyme responsible for melanin production is TYR. ROS buildup increases TYR activity, which may result in aberrant melanin synthesis. Excessive melanin accumulation may ultimately lead to hyperpigmentation disorders [37,38]. The catalytic action of TYR involves two distinct steps. L-DOPA is first created by hydroxylating L-TYR. Subsequently, L-DOPA is oxidized to dopaquinone, which undergoes several enzymatic steps to form melanin [39]. There are several ways to accomplish skin whitening, such as suppressing melanocortin 1 receptor (MC1R) or TYR activity, interfering with melanosome maturation and transfer, and inhibiting microphthalmia-associated transcription factor (MITF) [40]. Because of its strong whitening effects, TYR inhibitors are the most commonly used tactic in the cosmetics industry.

The long-term use of commercial TYR inhibitors, including KA and arbutin, is limited by safety concerns, such as cytotoxicity, skin irritation, and potential genetic damage, despite their effective inhibition of TYR activity [41]. This limitation has prompted researchers to explore natural alternatives. Importantly, a compound qualifies as a true enzyme inhibitor only if it specifically binds to the enzyme and reversibly reduces its catalytic activity, which necessitates rigorous characterization of the enzyme-inhibitor interaction [42]. Seaweed-derived TYR inhibitors have strong depigmenting action and good biosafety. They are becoming a new tactic to meet the needs of sustainable cosmetic development and to replace conventional synthetic whitening chemicals.

The most abundant source of strong TYR inhibitors is brown algae. Their profusion of phlorotannins, a family of polyphenolic chemicals unique to brown algae, is the primary cause of this trait [43]. Phlorotannins possess abundant phenolic hydroxyl groups, which confer significant metal-chelating capacity and antioxidant activity. By binding to the TYR active site, these hydroxyl groups can prevent excessive pigmentation and inhibit melanin formation [44]. The present study demonstrates that SFP inhibits TYR in a dose-dependent and reversible manner. Compared to irreversible inhibitors, reversible inhibitors bind to the enzyme’s active site non-covalently. They reduce catalytic efficiency while preserving the enzyme’s structural integrity. This mode of action results in lower off-target toxicity, reduced risk of sensitization, and no permanent modification of the protein. Therefore, reversible inhibitors such as SFP exhibit superior safety and tolerability profiles for cosmetic applications [45].

To investigate the whitening efficacy of SFP at the cellular level, we used A375 cells, a well-established melanogenesis model that reliably mimics native melanin production. Our findings showed that SFP dramatically reduced intracellular TYR activity and melanin synthesis in A375 cells. These results validate SFP’s potential as a cellular TYR inhibitor and are consistent with its inhibitory action on TYR.

One of the main pathogenic causes of hyperpigmentation is oxidative stress. Cellular oxidative stress results from an imbalance between ROS generation and scavenging, thereby enhancing melanin formation in melanocytes and positively regulating TYR activity [46]. In the current investigation, SFP reduced intracellular ROS levels in A375 cells in a dose-dependent manner, suggesting its antioxidant potential. This reduction in ROS may suppress ROS-activated signaling pathways that promote melanin synthesis, thereby reducing melanin overproduction. In addition, biochemical assays indicated that SFP exerted a direct inhibitory effect on TYR activity, while cellular assays further supported its anti-melanogenic effect. Previous research has shown that polyphenolic compounds derived from the brown alga *Ecklonia stolonifera* lower reactive ROS levels in B16F10 melanoma cells, which is consistent with our findings [32].

The present study employed A375 human melanoma cells as an in vitro model for preliminary screening of anti-melanogenic activity. Although A375 cells retain basal expression of functional melanogenic proteins, including TYR, and have been widely used for this purpose, they remain a transformed cell line with signaling pathways, proliferation characteristics, and pigmentation regulation that differ from those of normal human melanocytes. Therefore, the findings obtained from A375 cells should be regarded as preliminary evidence of anti-melanogenic potential. Further validation in normal human melanocytes, co-culture models, or three-dimensional skin equivalents is warranted to substantiate the translational potential of SFP in cosmetic formulations.

The complexity of physiological conditions calls for in vivo models to verify the effectiveness and safety profile of active substances, even when in vitro research provides direct evidence of molecular mechanisms. Zebrafish, as a vertebrate model organism, shares similarities with humans in terms of organ systems and genomic sequences [47]. Genes involved in melanogenesis in zebrafish, such as the TYR gene, are homologous to those in mammals. This high degree of genetic similarity makes zebrafish a valuable experimental model for anti-melanogenesis research [48]. Furthermore, as melanin accumulates on the body surface during the early stages of zebrafish growth, this pigmentation process can be visualized microscopically without the need for complex experimental procedures [49,50]. Polyphenon 60, a polyphenol derived from green tea, has been shown in earlier research to prevent melanin formation in zebrafish embryos [51]. In vivo assessment of SFP’s depigmenting properties was conducted using zebrafish embryos. The results demonstrated that SFP possesses anti-melanogenic and TYR inhibitory activities in the zebrafish model. These findings align with cellular research conducted in vitro. The consistency between in vivo and in vitro results provides a more robust evidence base for developing SFP as an active ingredient in whitening cosmetics. This finding also addresses the research gap left by studies relying solely on in vitro models of *S. fusiforme*. Nevertheless, the absence of a formal sample-size power analysis may limit the precision of the observed effects.

Aging skin is a complex phenomenon that involves both internal and external factors. UV light is the main extrinsic factor that causes skin photoaging. It can induce various cutaneous disorders, including hyperpigmentation, wrinkle formation, and other manifestations of photoaging [52]. The epidermis absorbs most ultraviolet B (UVB) radiation, allowing very little to reach the dermis. In contrast, UVA destroys dermal fibroblasts, the primary cell type in the dermis, by directly penetrating the dermal layer [53]. A key mechanism underlying UV-induced skin photoaging is the dysregulation of ECM degradation [54]. The ECM fills the intercellular spaces and is essential for maintaining tissue integrity [55]. Collagen and elastin are the primary components of the ECM that provide the skin with structural support. About 90% of the body’s collagen is COL1, and elastin is essential for the skin’s suppleness.

UV radiation is widely recognized to upregulate matrix MMPs in fibroblasts. Specifically, UVA induces MMP expression and secretion [56], and overexpression of these MMPs accelerates ECM degradation. This process ultimately results in skin laxity, wrinkle formation, and impaired barrier function [57]. Therefore, inhibiting MMP overexpression and preserving the dynamic balance between ECM synthesis and degradation are pivotal strategies for mitigating skin photoaging.

To assess the anti-photoaging effect of SFP in vitro, a UVA-irradiated HDF model was established. Enzymatic activity assays demonstrated that SFP effectively inhibited collagenase and elastase. At the cellular level, pretreatment with SFP significantly enhanced HDF cell viability following UVA exposure, indicating a cytoprotective effect. At the molecular level, SFP modulated multiple parameters related to ECM metabolism, including HA production and the expression of COL1, elastin, and MMPs. Notably, SFP stimulated HA production and increased COL1 and elastin expression. HA, a significant part of the ECM, is crucial for preserving tissue hydration and structural integrity [58,59]. Furthermore, SFP significantly reduced the UVA-induced upregulation of MMP-1 and MMP-3. Because MMPs degrade collagen and elastin, this reduction may contribute to preserving ECM integrity. A similar protective effect on ECM integrity has been reported for Sargachromanol E, a compound derived from *Sargassum horneri*, which was shown to preserve collagen by inhibiting ROS production and MMP expression [60]. Collectively, these findings suggest that SFP may protect HDF cells against UVA-induced photoaging, potentially by modulating the balance between ECM synthesis and degradation.

It should be noted that the SFP obtained in this study is a polyphenol-enriched fraction rather than a single pure compound. Although XDA-7 macroporous resin chromatography effectively enriched the phenolic components, the final product remains a complex mixture. Consequently, which active components in SFP are truly responsible for its whitening and anti-photoaging activities remains to be elucidated. Future studies should focus on isolating and structurally characterizing individual active compounds from SFP to clarify their precise molecular targets.

## 5. Conclusions

The present study demonstrates that SFP is a natural bioactive agent with effects on both melanogenesis and photoaging-related parameters. In terms of skin whitening, SFP acts as a reversible TYR inhibitor, dose-dependently reducing melanin synthesis and TYR activity in both zebrafish embryos and A375 cells. Regarding anti-photoaging, SFP downregulates UVA-induced MMPs expression and effectively elevates the levels of key ECM components—including COL1, elastin, and HA—in HDF cells, which may contribute to alleviating photoaging damage. Collectively, these findings suggest that SFP exhibits both skin-whitening and anti-photoaging potential. However, further evaluations, including skin compatibility and long-term safety, are required to assess its potential as a cosmetic candidate ingredient.

## Data Availability

Data are available from the corresponding author upon reasonable request.
